# Java Simulations of Embedded Control Systems

**DOI:** 10.3390/s100908585

**Published:** 2010-09-15

**Authors:** Gonzalo Farias, Anton Cervin, Karl-Erik Årzén, Sebastián Dormido, Francisco Esquembre

**Affiliations:** 1 Department of Computer Science and Automatic Control, UNED, Juan del Rosal 16, Madrid 28040, Spain; E-Mail: sdormido@dia.uned.es (S.D.); 2 Department of Automatic Control, Lund University, Box 118, Lund SE-221 00, Sweden; E-Mails: anton@control.lth.se (A.C.); karlerik@control.lth.se (K.Å.); 3 Department of Mathematics, Murcia University, Murcia 30071, Spain; E-Mail: fem@um.es (F.E.)

**Keywords:** control education, embedded control systems, virtual labs, TrueTime, Easy Java Simulations

## Abstract

This paper introduces a new Open Source Java library suited for the simulation of embedded control systems. The library is based on the ideas and architecture of TrueTime, a toolbox of Matlab devoted to this topic, and allows Java programmers to simulate the performance of control processes which run in a real time environment. Such simulations can improve considerably the learning and design of multitasking real-time systems. The choice of Java increases considerably the usability of our library, because many educators program already in this language. But also because the library can be easily used by Easy Java Simulations (EJS), a popular modeling and authoring tool that is increasingly used in the field of Control Education. EJS allows instructors, students, and researchers with less programming capabilities to create advanced interactive simulations in Java. The paper describes the ideas, implementation, and sample use of the new library both for pure Java programmers and for EJS users. The JTT library and some examples are online available on *http://lab.dia.uned.es/jtt*.

## 1. Introduction

As computers become smaller, faster, and cheaper, their application scope increases to reach almost every aspect of modern life. In earlier times, computers were used by scientists to solve just a simple equation in execution times that lasted as much as needed. But nowadays they are present in practically any scientific and technological field, controlling as many processes as possible, and with an increasing demand of performance [1]. One important practical application of computing is that of embedded control systems. An embedded control system consists of the use of a dedicated computer whose main task could be to apply a control algorithm in order to keep a signal from a piece of equipment or a process inside prescribed safety margins, despite disturbances. The control task typically executes periodically and under limited implementation resources (CPU time, communication bandwidth, energy, memory. . .). If the limited resource is the CPU time, then the system is generically called a real-time system.

Academic interest in real-time systems and in control theory have both a long, but separated, tradition. Research on real-time scheduling dates back to the beginning of the 1970s, and nowadays reaches far even into unconventional areas of application on industry [2–4]. However little of this work has focused on control tasks. On the other hand, digital control theory, with its origin in the 1950s, has not addressed the problem of shared and limited resources in the computing system until very recently [5,6]. Instead, it is commonly assumed that the controller executes as a single loop in a dedicated computer.

Typically, the control engineer does not know (or care) about will happen in the implementation phase of the control algorithm. The common assumption is that the computing platform can provide periodic sampling and the computation delay of the controller is either negligible or constant. Reality tends to be far different. Today, processors are built with caches and pipelines, software is divided into several modules, signals need to be communicated through networks, and there is a strong trend towards the use of commercial, off-the-shelf (COTS) hardware and software. These factors contribute to make the time response of the computing platform, which is shared among many tasks, unpredictable.

On the other side of the problem, the computer engineer who implements the control system can also make wrong assumptions. It is commonly assumed that controllers have a fixed execution-time, that all control loops are periodic, or that controllers deadlines are critical.

In reality, many control systems have varying execution time demands, such as model predictive controllers. Besides, some control systems are not sampled against time, such as the combustion engines controllers or the use of event-based control schemes, where the existence of traditional periodic sensors is replaced by send-on-delta [7] strategies in order to optimise (in terms of economic or energy costs) the exact moment when signals have to be sampled. Finally, in many situations a single missed deadline in a control system does not necessarily cause system failure.

This misunderstanding between both types of engineers is now been addressed by an emerging interdisciplinary approach, where control and real-time issues are discussed at each design level. The development of algorithms for co-design of control and real-time systems requires new tools, one of the most successful being the freeware Matlab toolbox TrueTime [8,9]. However, this tool requires Matlab/Simulink [10] to carry out the simulations, which limits its use to Matlab users. In order to make the study of embedded control systems possible for a wider audience, we have implemented an Open Source Java library, which we call JTT (Java TrueTime) [11]. This Java library uses the key concepts and architecture of the TrueTime toolbox to make the simulation of embedded control systems easier for Java programmers. Besides, typically simulations created with JTT present a better performance to those developed with TrueTime. Simulation of wired and wireless networking features such as described in [12] and in TrueTime [13] are not yet implemented.

**Figure 1.**
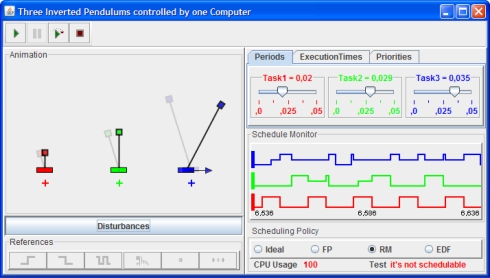
A virtual lab built with EJS and JTT. Three inverted pendulums controlled by three periodic controllers running on the same computer.

We chose Java as the implementation language because it is currently one of the most popular programming languages. This is specially true in the educational world, which is benefiting noticeably from the pedagogical advantages of the use of computer simulations in the learning process [14–16]. Moreover, because some control educators find it difficult to program a simulation in plain Java, we designed the library so that it is easy to use with Easy Java Simulations (EJS) [17,18]. EJS is an open source modeling and authoring tool that greatly facilitates the creation of advanced simulations in Java with high-level graphical capabilities and an increased degree of interactivity. Figure 1 shows a sophisticated simulation of an embedded control system for three inverted pendulums created with EJS and our library JTT.

The rest of this paper is organized as follows. Section II revises the main concepts and design problems of embedded control systems. Section III introduces the library, and discusses its Application Programming Interface (API) and its implementation. In Section IV a complete Java example shows how the library is used in the simulation of a simple DC servo system controlled by an embedded PID controller. Section V shows how the JTT library is used in the creation of an EJS simulation (with an advanced visualization) of the same DC servo system. Finally, Section VI presents the main conclusions and discusses future work.

## 2. Embedded Control Systems

In an embedded control system, the (usually multiple) tasks are normally executed in what is called *real-time*. A system is said to be real-time if the total correctness of the operation depends not only upon its logical correctness, but also upon the time in which it is performed [1]. Real-time systems can be classified in two subcategories: *hard* real-time systems, in which the completion of an operation after its deadline may lead to a critical failure of the complete system and *soft* real-time systems, which tolerate such lateness and may respond with decreased service quality (such as a longer settling time).

A simple example is that of stabilizing an inverted pendulum (see Figure 1) by moving its base back and forth (the academic version of the how the Segway Personal Transporter keeps its verticality). Suppose our operation requirements specify that the pendulum must recover its verticality as soon as possible after suffering any moderate perturbation. If the sampling period of the vertical angle of the pendulum is 80 ms, with a time delay of 20 ms for the engines to act on the base, a reasonable design could require that the control algorithm is executed every 80 ms and has a worst case execution time of 60 ms. For the system to avoid the pendulum from falling, the control algorithm must be both correctly designed and must be applied in time.

Real-time tasks such as our control of verticality can be periodic, aperiodic, or sporadic, and are characterized by different parameters, among which are:

- **release time:** which indicates the next instant in time when a task should be executed.
- **finish time:** to indicate when a task has finished its execution.
- **execution time:** which is the duration of the task execution.
- **period:** to indicate the amount of time at which a periodic task has to be released. When the task is periodic, the release time is always a multiple of the period.
- **deadline:** which indicates the maximum allowed execution time to ensure correct execution. It is common to take the period as the deadline of a periodic task.

Typically a control task executes in parallel with several other tasks, including other control tasks. This puts focus on the *scheduling policy* of the system, which is the algorithm that decides which task to execute at a given time. The presence of a scheduling policy introduces a new parameter for a task, its *priority* or preference with respect to other tasks in the system.

In our example above, the control of the pendulum’s verticality would typically be a top-priority, periodic task with a period of 80 ms and execution time smaller than 60 ms, which makes a deadline of 80 ms reasonable. In cases where there are more tasks competing for CPU resources, a smaller deadline could be prescribed.

**Figure 2.**
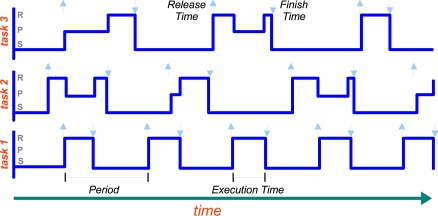
Schedule plot: three periodic tasks are running on the same CPU, tasks 1 and 3 have the highest and lowest priority respectively. Up arrows in a task plot indicate the released times of that task, down arrows indicate the task finish times. The initials R, P, and S indicate the possible states of the tasks. Note that the task 1 is never pre-empted.

Under a scheduling policy tasks may be in one of the three following states: *running*, *preempted* or blocked and *sleeping*. Running means that the task is actually executing. Preempted means that the task needs to be executed, but it is not being executed because another task is running (usually one with higher priority). Sleeping indicates that the task has finished and is waiting for its next release time. A schedule plot such as the one shown in Figure 2 is a graphical used to illustrate the evolution of the states of the tasks in time.

The scheduling policy can be static or dynamic. For instance, Rate Monotonic (RM) is a popular static scheduling policy that assigns the priorities of the tasks on the basis of their period: the shorter the period, the higher the priority of the task. Earliest Deadline First (EDF) is a dynamic scheduling policy that places tasks in a priority queue. Whenever a **scheduling event** occurs (a task finishes, a new task is released, *etc.*) the queue is searched and the process closest to its deadline is scheduled for execution.

## 3. Simulating an Embedded Control System

Given the variability of practical situations, the designer of an embedded control system needs to think carefully and test the possible configurations of the tasks and their parameters. Setting correct priorities and configuring the tasks appropriately can have a great impact on the total correctness of the system. In particular, the control tasks can be split into *code segments*, so that only a part of the control algorithm is applied during each periodic execution of the task.

Also, during the test process, the designer may want to play with the actual execution times of the tasks in order to test the robustness of the control to variations caused by the CPU being involved in other processes. The final goal is to design a control algorithm that meets the needs and resists the variations caused by the actual implementation and unpredictable real situations.

**Figure 3.**
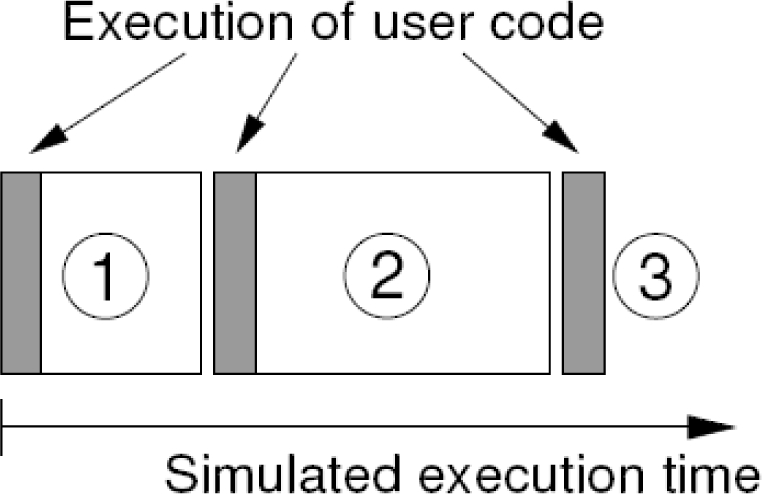
TrueTime code model. The execution of task code (or user code) is modelled by a sequence of code segments that are executed in sequence by the kernel.

Inspired by the successful ideas and architecture of the TrueTime toolbox for Matlab, we created our JTT Java library to simulate code execution and scheduling of tasks in a real time environment. The library allows converting a Java simulation of a control process into an embedded control system by defining one or several kernels (which simulates a computer) that execute tasks according with a given scheduling policy, including RM and EDF. Similar to TrueTime, the code of a task is divided into segments as shown in Figure 3. The kernels, tasks and policies are also highly configurable. Since JTT is a Java library, the systems can be implemented using pure Java code. The programmer creates and adds the system components using the library’s API, and the library manages automatically all the internal data structures and organizes the execution of the tasks. Non-programmers can use the library via Easy Java Simulation, as discussed in Section V.

### 3.1. JTT’s Application Programming Interface

**Figure 4.**
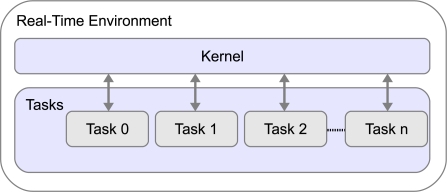
Main classes in the JTT package.

The JTT package provides four public classes and one abstract class. The three most important are: *RTenv*, *Kernel* and *Task* (see Figure 4).

A real-time Java environment is an object of the public class *jtt.RTenv*, which provides the basic functionality for implementing of the real-time environment. *jtt.RTenv* is a singleton class that can not be instantiate from another class. A kernel is an object of the class *jtt.Kernel* that simulates a computer which can execute one or more tasks. Kernels are instantiated using the constructor: **Kernel();**.

Kernels are added to the real-time environment using the static method: **RTenv.addKernel(Kernel kernel);**. A task is obtained by using the public constructor of *jtt.Task* class: **Task();**.

Tasks can be later customized using standard setter and getter methods. Following TrueTime’s code model, tasks in the JTT library are divided into code segments. A code segment is an object, programmed by the user that extends the *jtt.CodeSegment* abstract class. Segments can also be added or removed from the task after instantiation using convenience task methods.

This object-oriented structure provides a flexible and powerful way to create sophisticated tasks. Alternatively, for simple situations, the task’s code can be also defined by using reflection. This option can use suitable methods to run the code of the task. The method can include calls to the convenience static method **RTenv.endSegment(double time)** to divide the code of the method into code segments.

With this information, the kernel object manages two internal queues to control the execution of the tasks. The first queue is sorted by priority and keeps the identifiers of tasks which are ready to be executed by the kernel. The second queue is sorted by release time and keeps the identifiers of tasks which are waiting to be released. The kernel uses this task to determine the release time of the next task and to execute the task segments according to the scheduling policy.

Authors can use this API to modify an existing simulation which they can step in time. They can modify their initialization to create and add the required kernels and tasks, and then the main loop to request the time of the *scheduling event* of the closer task in all kernels. If this time is closer to the desired step, the program passes over the control of the execution to the kernel to execute the task code.

The classes *Kernel* and *Task* are implemented using Java threads. This implementation choice allows interrupting the execution of a task and restart it when it is next released. To coordinate the execution of the tasks, each kernel object has an object of the private class *jtt.Token*. When the kernel receives the request to run, it gives its token to the task that is to be released. The task returns the token to the kernel when it finishes the execution of a code segment and the kernel returns the control to the calling program.

### 3.2. Sample Implementation

To exemplify briefly this structure, let’s suppose the original program consists of the following, rather simplistic pseudo-code shown in Listing 1.

**Listing 1.** Original simulation

1	**public class** MyProcess {
2	*// Initializes the process*
3	**public** MyProcess ( ) {
4	. . .
5	}
6	*// Steps the process for an increment of time*
7	**public void** step (**double** dt) {
8	. . .
9	}
10	*// Simulate the process*
11	**static public void** main (String [] args) {
12	**double** time = 0, tFinal = 1.0, dt = 0.001;
13	MyProcess process = **new** MyProcess ( );
14	**while** (time <tFinal) {
15	process . step (dt);
16	time += dt;
17	*// output process variables*
18	. . .
19	}
20	} *// end of class*

To convert this process into an embedded control system, the programmer needs to modify this class as the Listing 2 shows. Here some API methods for creation and configuration of the tasks and kernels were used. Note how the simulation of the embedded process is done in the static method *main*. Next subsection will discuss this in detail.

**Listing 2.** Modified simulation, version 1.

1	**import** jtt.*; *// Import the JTT package*
2	**public class** MyProcess {
3	*// Initialize the process*
4	**public** MyProcess ( ) {
5	. . .
6	*// create a task*
7	Task task1 = **new** Task ( );
8	task1.setPeriod (0.08); *// period 80 ms*
9	task1.setPriority Value (0) *// Top priority*
10	*// add code to a task*
11	task1.addCode (**new** CodeSegment ( ) {
12	*// code of the first segment*
13	**public double** code ( ) {
14	. . .
15	**return** 0.03; *// execution time*
16	}
17	});
18	. . .
19	*// create another task*
20	Task task2 = **new** Task ( );
21	task2.set Period (0.1); *// period 100 ms*
22	task2.set PriorityValue (1) *// priority*
23	*// add code to another task*
24	. . .
25	*// create kernel and add it the tasks*
26	Kernel kernel = **new** Kernel ( )
27	kernel.setScheduling Policy (Kernel.FP);
28	kernel.addTask (task1);
29	kernel.addTask (task2);
30	*// add the kernel to the real time environment*
31	RTenv.add Kernel (kernel);
32	}
33	*// Step the process for an increment of time*
34	**public void** step (**double** dt) {
35	. . .
36	}
37	*// Simulate the process*
38	**static public void** main (String [] args) {
39	**double** time = 0, tFinal = 1.0, dt = 0.001;
40	MyProcess process = **new** MyProcess ( );
41	**while** (time <tFinal) {
42	**double** nextEvent = RTenv.nextEvent ( );
43	**if** (nextEvent <time + dt) {
44	process.step (nextEvent−time);
45	RTenv.runKernel ( );
46	time = nextEvent;
47	} **else** {
48	process.step (dt);
49	time += dt;
50	}
51	*// output process variables*
52	. . .
53	}
54	} *// end of class*

The Listing 2 uses one way to add code to the tasks, *i.e.*, overriding the class *CodeSegment*. However, as we said before, there is another way much more simple that can be preferred by non-programming authors. That way is called reflection.

The using of reflection provides to beginner programmers an easy way for adding code to a task. Listing 3 shows how it can be done. Observe that reflection is chosen because we have added at the beginning of the process’ initialization the method *setReflectionContext*. The input parameter of this method is used to define the Java object where actually the code exist, in this case in the same class *MyProcess*. Adding code to a task is done by using the method *addCode*, where the input parameter sets the name of the Java method that has to be run when the task is executed. Note how the method *endSegment* is used to split the task’s code in code segments. The input parameter of *endSegment* is used to return the execution time of a code segment.

**Listing 3.** Modified simulation, version 2.

1	**import** jtt.*; *// Import the JTT package*
2	**public class** MyProcess {
3	*// Initialize the process*
4	**public** MyProcess ( ) {
5	*// Sets reflection*
6	RTenv.setReflectionContext (**this**);
7	. . .
8	*// create a task*
9	. . .
10	*// add code to a task*
11	task.addCode (“mycode”);
12	*// create kernel and add it the tasks*
13	. . .
14	}
15	. . .
16	*// code of a task*
17	**public void** mycode ( ) {
18	*// code of the first segment*
19	. . .
20	RTenv.endSegment (0.3); *// execution time*
21	. . .
22	*// code of the last segment*
23	. . .
24	RTenv.endSegment (0.1); *// execution time*
25	}
26	} *// end of class*

Both ways for adding task’s code can be used indistinctly in the most of cases. However, reflection could be easier to use for beginner programmers, whereas that overriding the class *CodeSegment* could be much more useful if the task’s code is modified at runtime.

### 3.3. Integration of JTT in Advanced Simulations

In general, the simulation of an embedded control system consists of two main parts: the computer and the physical system. First part simulates a computer (*i.e.*, a kernel) where the control task is executing, while the second part simulates the model of the physical system or the process to be controlled. As we have seen, the JTT package allows authors to simulate the computer behaviour but, the simulation of the physical system has to be provided by authors, who have to write the required Java code or to use other suitable Java packages or tools like EJS.

Normally physical systems are modelled using Ordinary Differential Equations (ODE), for that reason typical simulators have various ODE solvers (also called, numerical or integration methods) to simulate these ODE models. The implementation in Java of a simple ODE solver simulator should not be a difficult labour for even a beginner Java programmer, in fact, there are many open source code available on Internet [19] (e.g., *http://www.opensourcephysics.org*). However probably, to write all the Java code required for creation of highly visual and interactive simulations could be a hard (or at least a time consuming) task.

Considerer for instance, the ODE model given by (1). In this system the derivatives are given by the function *f*() and the initial state of the system is represented by *x_n_*. To solve an ODE model means to advance the system from an initial state *x_n_* to a final state *x_n_*_+1_. The experimented reader have surely noted that this action is implemented by the method *step()* in our sample simulation of Listing 1. There are many solvers for ODE models, one of the most popular ODE solver is the Runge-Kutta fourth-order. This algorithm calculates the final state *x_n_*_+1_ by means a weighted average given by (2). This approximation is fifth-order accurate in the step size for a single step. The constants *k*_1_, *k*_2_, *k*_3_ and *k*_4_ represent the derivative at beginning and middle time. The step size corresponds to the interval time between the initial and final time.

(1) $$\dot{x}=f\left(t,x\right),\;\;\;x\left(t_{n}\right)=x_{n}$$

(2) $$x_{n+1}\approx x_{n}+\frac{k_{1}+2k_{2}+2k_{3}+k_{4}}{6}$$

**Figure 5.**
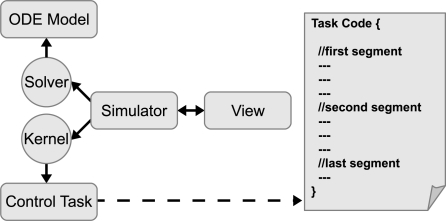
Diagram of an embedded control system simulation with JTT. Kernel simulation is provided by JTT, Solver and View have to be programmed or facilitated by other Java tools like EJS.

The coordination of both elements, kernel and solver, can be easily done by a simulator just executing repeatedly the ODE solver and the kernel at specific times, see Figure 5.

When both solver and kernel are executed by the simulator, they internally set their next time to be called by the simulator. In the case of the solver this next invocation time is the next integration step determined by the algorithm that implements the solver. In the case of the kernel, the next invocation time is based on the time of the next **scheduling event**. An event in the kernel can be for instance a task that has finished a code segment, or a task that was sleeping and should be released.

Obviously, the simulator runs the system at the time given by the minimum of both next invocation times. Note that when when the kernel has to be invoked, the simulator has also to call to the solver in order to get the state of the ODE model at that time. Note also that the control execution of the kernel is done by the primitive *runKernel()*, while the method *nextEvent()* has to be used in order to get the next scheduling event of the kernel. These ideas about the kernel and solver integration, are clearly exposed in the static method *main* of our modified simulation of Listing 2.

## 4. JAVA-JTT Approach

In this section we present a virtual lab of an embedded control system. First, the model of a DC servo motor is presented. Then, the Java code to simulate the system is commented. The objective of this virtual lab is just to show how to create the simulations of embedded control systems using Java and the JTT library.

### 4.1. Embedded Control of a DC Servo

In this subsection we introduce a DC servo system controlled by an embedded PID controller [20]. The pedagogical purpose of the virtual lab is to show how the execution time of the controller induces a delay in the feedback loop that might deteriorate the performance.

The embedded system consists of a periodic task controlling a simple DC servo system. The physical system to be controlled is modelled as an ODE given by (3) and (4).

(3) $$\left[\begin{array}{c} {\dot{x}}_{1}\\ {\dot{x}}_{2} \end{array}\right]=\left[\begin{array}{cc} 0 & 1\\ 0 & -1 \end{array}\right]\;\left[\begin{array}{c} x_{1}\\ x_{2} \end{array}\right]+\left[\begin{array}{c} 0\\ 1000 \end{array}\right]\;u$$

(4) $$y=\left[\begin{array}{cc} 1 & 0 \end{array}\right]\;\left[\begin{array}{c} x_{1}\\ x_{2} \end{array}\right]$$

The controller is described by a periodic task divided in two subtasks or code segments. First code segment computes the control action using a PID algorithm. Second code segment takes the computed control action and sends out this signal to the servo system. Since the first subtask consumes much more time from CPU than the second one, we can assume that only first code segment spends time.

### 4.2. Simulation of the Embedded Servo in Java

For simplicity, the simulation of the servo motor follows the same structure presented in Listing 2. However, also two new methods are added to this listing: *getRate(double[] state, double[] rate)* and *step(double dt)*. Part of the new modified simulation is presented in the Listing 4.

**Listing 4.** Modified simulation, version 3.

1	**import** jtt.*; *// Import the JTT package*
2	**public class** MyProcess {
3	*// Initialize the process*
4	**public** MyProcess ( ) {
5	*// create a task (a PID controller)*
6	Task task = **new** Task ( );
7	task.setPeriod (0.012); *// period = 12 ms*
8	*// add code to task*
9	task.addCode (**new** CodeSegment ( ) {
10	**public double** code ( ) {
11	controlAction = calculate (reference, output);
12	**return** 0.02; *// 20 ms*
13	}
14	});
15	task.addCode (**new** CodeSegment ( ) {
16	**public double** code ( ) {
17	input = controlAction;
18	**return** 0; *// 0 ms*
19	}
20	});
21	*// create kernel and add it the task . . .*
22	}
23	*// Gets rate of the ODE model*
24	**public void** getRate (**double** [] state, **double** [] rate) {
25	rate [0] = state [1];
26	rate [1]= − state [1] + 1000 * input;
27	rate [2] = 1;
28	}
29	*// Step the process for an increment of time*
30	**public void** step (**double** dt) {
31	getRate (state, rates1);
32	**for** (**int** i = 0; i <numEqn; i++)
33	k1 [i]= state [i]+ stepSize * rates 1 [i]/2.0;
34	getRate (k1, rates 2);
35	**for** (**int** i = 0; i <numEqn; i++)
36	k2 [i]= state [i]+ stepSize * rates 2 [i]/2.0;
37	getRate (k2, rates 3);
38	**for** (**int** i = 0; i <numEqn; i++)
39	k3 [i]= state [i]+ stepSize*rates3 [i];
40	getRate (k3, rates4);
41	**for** (**int** i = 0; i <numEqn; i++)
42	state [i]= state [i]+stepSize*(rates1[i]+2*rates2[i]+2*rates3[i]+rates4[i])/6.0;
43	}
44	*// Computes the PID*
45	**public double** calculate (r, y) {
46	P = Kp*(beta*r−y); I = Iold; D = Td/(N*h+Td)*Dold+N*Kp*Td/(N*h+Td)*(yold−y);
47	Iold = Iold + Kp*h/Ti*(r−y); Dold = D; yold = y;
48	**return** (P + I + D);
49	}
50	*// Simulate the process*
51	. . .
52	} *// end of class*

The method *getRate(double[] state, double[] rate)* is shown in Listing 4 line 23. This method is used to describe the servo as an ODE, and also to update the derivatives (or rates) given by (3). The ODE model of the DC servo has two states (*x*_1_ and *x*_2_), but, for practical reasons, we consider the time as other state of the model. So for our convenience, the servo has three states (*x*_1_, *x*_2_ and *time*). The states are coded by the array of doubles *state*, where *x*_1_ is *state[0]*, *x*_2_ is *state[1]* and *time* is *state[2]*. Note that the rate of the third state (time) is computed in the last rate.

Second method, *step(double dt)*, is added to step the process for an increment of time (*dt*). This method, see Listing 4 line 29, implements the Runge-Kutta algorithm to solve the ODE model of the servo. The method uses the variable *state* and the method *getRate* defined previously.

The task code of the PID controller is added in the simulation in listing 4 line 8. In the first segment, the task gets the control action (by calling to *calculate*), which is used in the second segment to feed the input of servo. Variables *reference*, *input* and *output* represent the reference, the input and the output of the process. Note that the code was added overriding the class *CodeSegment*.

The rest of the simulation is similar to the first modified version (see Listing 2). Although this version is quite simple, just to show how to use the JTT package in Java, authors with programming skills could use the Java packages *awt* and *swing* to create a more visual and interactive version, only adding a few lines of code to this simulation (see an example in *http://lab.dia.uned.es/jtt*).

## 5. EJS-JTT Approach

Here we present two examples using JTT library in EJS. The first example is the same simulation of the previous embedded control system but, using now JTT and EJS. This option could be speciality useful for authors who prefer to use all the facilities provided by EJS to build simulations with a high level of interaction and visualization. The second example describes some details about the simulation of the three inverted pendulums shown in Figure 1.

### 5.1. Simulation of the Embedded Servo in EJS

In EJS, every application is divided in two main parts: the *Model* and the *View*. In the *Model* we initialize the embedded system, and also we describe the ODE model and the code function of the task. In the *View* we use the visual elements of EJS to build the GUI of the simulation. Before starting with the code, we have to import the JTT package and to declare the variables. To import the JTT package, EJS provides of a special dialog window to browse the file *jtt.jar* and to enter the corresponding import statement. To declare the variables, we have to move to the section *Variables* and there, the kernels and tasks variables have to be defined.

In Listing 5 the script for *Initialize the Embedded System* is shown. This script is written in the section *Initialization* in EJS. Note that the code for *Initialize the Embedded System* is almost the same that the code shown in previous listings. However here, we specify that the schedule data must be available for plotting purposes. This is done using the methods *setSchedule* and *setScheduleWindow*. The second method allows to define the time extension of the schedule data available.

Regarding to the task code, the reader can see that the reflection was selected as a way to implement the embedded system. As Listing 3, the method *setReflectionContext* defines that *this* is the object where the code function of the task is located. This should be the general situation in EJS, since methods defined by users, like the code function *taskcode*, are normally located in the section *Custom*. Obviously the other way for adding task’s code is also possible in EJS.

**Listing 5.** Creation of the kernel and task in EJS.

1	*// Initialize the Embedded System*
2	RTenv.setReflectionContext (**this**);
3	kernel =**new** Kernel ( );
4	kernel.setSchedule (**true**);
5	kernel.setScheduleWindow (0.5);
6	task =**new** Task ( );
7	task.setPeriod (0.012);
8	task.addCode (“taskcode”);
9	kernel.addTask (task);
10	RTenv.addKernel (kernel);

Listing 6 shows the method that implements the code function of the *taskcode*. Note that *taskcode* is quite similar to the previous version of the simulation. However, since we are using reflection, the task code is divided now into code segments using the method *endSegment*. Input parameter of *endSegment* represents the execution time of the code segment as it has been said. Other user methods like *calculate*, which returns the computed control action, are also implemented in the section *Custom*.

**Listing 6.** The method ”taskcode” used by the periodic task

1	**public void** taskcode ( ) {
2	*// Update Output*
3	output =x1;
4	*// Calculate Action*
5	controlAction = calculate (reference, output);
6	RTenv.endSegment (executionTime);
7	*// Send Out Control Signal*
8	input = controlAction;
9	RTenv.endSegment (0);
10	}

In the section *Evolution* of the *Model* we have to write all the code that should be executed continuously by the simulation, *i.e.*, the solver and kernel events, and also the plotting of the schedule data.

**Figure 6.**
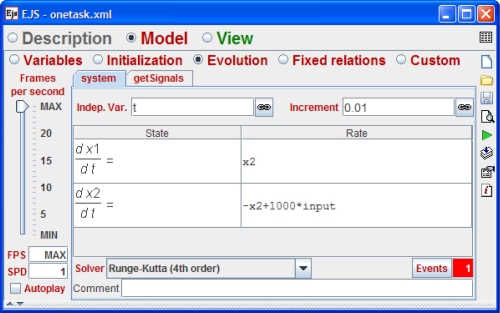
Ordinary differential equations of the model system using the editor of EJS. The ODE models are defined in the section *Evolution* in EJS.

Regarding to the solver and the plant dynamics, EJS provides an ODE editor to describe the ODE model given by (3) (see Figure 6). There are many solvers available in EJS, including the Runge-Kutta algorithm commented previously in Section 3.

EJS has also a way to detect events using the method of bisection. This feature will be useful in our case for detection of scheduling events. The events in EJS are adding by pressing the *Events* button (see Figure 6) and defining two parts. First part represents the called *zero cross function*, which has to return zero when the event has to be triggered. The second part of the event represents the *action* of the event, which is a set of statements that has to be executed when an event is triggered. Take into account that solvers in EJS always update the state of the ODE model before to call any event.

Four our purposes, the detection of the kernel event is quite simple. The *zero cross function* is just the remaining time to the next scheduling event, *i.e.*, *return RTenv.nextEvent(t);*. The *action* is also simple, because the only one statement needed to call the corresponding kernel is *RTenv.runKernel()*;.

To capture the schedule data the Listing 7 is also put on the section *Evolution*, but in other *evolution page* called *getSignals* (see Figure 6). This script uses the method *getSchedule* to get schedule data (arrays *time* and *value*) of the task. This data will be used by a *polygon* (a visual element of EJS) to plot the schedule state of the task in the GUI of the simulation.

**Listing 7.** Getting the schedule data of the task.

1	. . .
2	// *******Capture Schedule Signals******
3	taskSchet=task.getSchedule (“time”);
4	taskSchev=task.getSchedule (“value”);
5	points = taskSchev.length;
6	. . .

**Figure 7.**
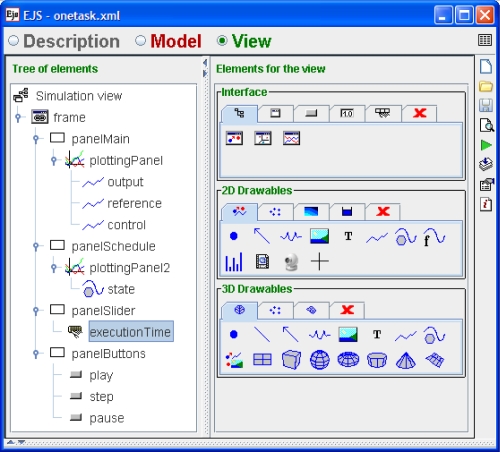
Section View of the EJS. Elements on the right are provided by EJS to build the tree-like structure on the left, which describes the GUI of the simulation of Figure 8.

After to implement the code in the *Model*, we have to use the visual elements provided by EJS to build the GUI of the simulation. There are good references that describe very well this action in detail [18], for that reason we just show in the Figure 7 the final result of using the visual elements of EJS. Four kind of elements are quite important in this view. The *PlottingPanels* used to show the axis of coordinates. The *traces: output*, *reference* and *control*, used to plot the output, control and reference signals of the system. The *polygon state* used to graph the schedule data. And finally the *slider executionTime* which allows end users to modify the execution time of the first code segment of the controller (see Listing 6). Note that the exchange of data between the real-time tasks and the GUI is done when either a solver step or a kernel event is executed.

**Figure 8.**
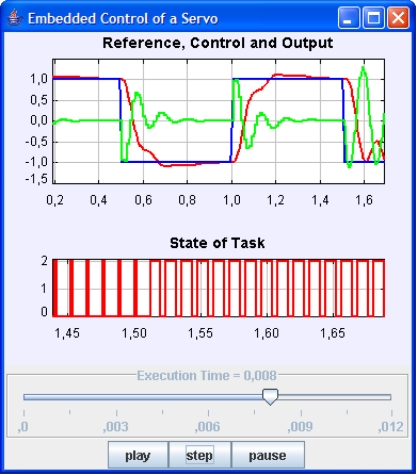
GUI of the virtual lab developed using the JTT-EJS approach.

The GUI of the simulation is shown in Figure 8. The virtual lab has two plots. Upper plot shows the signals reference, control and output of the system. Bottom plot, presents the schedule data of the task. There is also a slider to control the execution time and three buttons to control the simulation. In the GUI we can see how the increasing of execution time of the controller affects negatively to the control performance. This can be notice in the GUI, because the execution time has been changed from 2 ms to 8 ms at 1.5 s.

### 5.2. Control of Three Inverted Pendulums

This virtual lab simulates a more advanced example than the previous ones. In this case, three inverted pendulums of different lengths should be controlled by a computer with limited computational resources [6]. Control objectives of the system are to reach a desired position for the cart, while the pendulum keeps its verticality. A linear digital controller is designed (by state-space method) for each pendulum [21,22]. The pendulum lengths motivate different periods for the three controllers. Other parameters such as control gains or execution times should be similar in all cases.

As the previous example, the control task is divided in two code segments, one segment to compute the control action (given by a state feedback control strategy) and another one to send out, to each pendulum, the computed control action.

The GUI of this virtual lab is shown in Figure 1. On the left side, an animation of the three inverted pendulum is presented. Students can select different types of references, modify manually desired position points and even apply disturbances to the angle of the pendulums. At the right side, users can modify the parameters of all tasks such as, period, execution time and priority. There is also possible to select one of the three scheduling policies among other options.

Main goal of this virtual lab, from the pedagogical point of view, is to show how the scheduling policy affects the control performance. For instance the Figure 1 shows the state of the pendulums two seconds later that a moderate disturbance was apply. Note that two pendulums have totally recovered the verticality, however the largest pendulum is still trying to stabilize. This fact, is a consequence of the selected scheduling policy. As the Rate Monotonic (*RM* at the GUI) was selected, the task’s priorities are sorted by the period. The largest pendulum has the largest period and so, the lowest priority. This introduces variables delays in the execution of the controller due to the interruption of the other two pendulums (see the schedule plot at the GUI). If the scheduling policy is changed to Earliest Deadline First (*EDF* at the GUI), the CPU is shared between the task in a more fair way, and so the verticality of all pendulums can be achieved approximately at the same time. More details about the system can be found in [6].

## 6. Conclusions and Further Work

Control theory and real-time systems have both a long, but separated, tradition. Typically, an embedded control system have been implemented in two unconnected phases by two kind of engineers: a control engineer and a computer engineer. Both engineers have made wrong assumptions about each phase. Computation delay of the controller is negligible or controller deadlines are always critical, are wrongly assumed as true.

This misunderstanding have required of new simulation tools to study the real-time control systems. One of them is the successful Matlab-based toolbox TrueTime. However the toolbox is limited to Matlab users, which can be an important restriction for many students. For that reason, we have created a library called JTT, which have been implemented in Java to take advantages of this language specially from the pedagogical point of view. Interactivity and rich graphical contents can be also added to these simulations in order to help specially to the learning process of embedded control systems.

Non-programming instructors, who are not use to Java, can still build simulations by using the JTT library with Easy Java Simulations. This approach facilitates enormously the creation of simulations with a high degree of interactivity and visualization.

To show how to use the library JTT, three virtual labs have been presented in the article, one built completely in Java, and the others two by using Easy Java Simulations. Some important effects, about the real-time parameters in the embedded control systems are also discussed in that simulations. More examples can be found in *http://lab.dia.uned.es/jtt/*.

The framework for embedded control simulations is still under developing, and to switch from the simulation environment to the real implementation is not direct and easy. Many work in this sense is still required, so further work could involve the development of new functionalities to, for instance, interact properly with data acquisition cards in order to control real equipment from real-time tasks. Other further work could be the supporting of wired and wireless communication networks between the kernels of the real-time environment.

## References

[1] Burns A, Wellings A (2001). RealTime Systems and Programming Languages.

[2] Nolte T, Passerone R (2009). Guest Editorial Special Section on Real-Time and (Networked) Embedded Systems. IEEE Trans. Ind. Inf.

[3] Buttazzo G, Kuo TW (2009). Guest Editorial: Special Issue on Real-Time Systems Part II. IEEE Trans. Ind. Inf.

[4] Ma L, Xia F, Peng Z (2008). Integrated Design and Implementation of Embedded Control Systems with Scilab. Sensors.

[5] Cervin A, Henriksson D, Lincoln B, Eker J, Årzén K (2003). How does Control Timing Affect Performance?. IEEE Contr. Syst. Mag.

[6] Cervin A (2003). Integrated Control and Real-Time Scheduling.

[7] Miskowicz M (2006). Send-On-Delta Concept: An Event-Based Data Reporting Strategy. Sensors.

[8] TrueTime’s home page. http://www.control.lth.se/truetime (accessed on 2 September 2010).

[9] Ohlin M, Henriksson D, Cervin A (2007). TrueTime 15 Reference Manual, Manual.

[10] The Matworks, Matlab’s home page. http://www.mathworks.com. (accessed on 02 September 2010).

[11] Farias G, Cervin A, Årzén K, Dormido S, Esquembre F Multitasking Real-Time Control Systems in Easy Java Simulations.

[12] Xia F, Tian Y, Li Y, Sung Y (2007). Wireless Sensor/Actuator Network Design for Mobile Control Applications. Sensors.

[13] Andersson M, Henriksson D, Cervin A, Årzén K Simulation of wireless networked control systems.

[14] Heck BS (1999). Special Report: Future Directions in Control Education. IEEE Contr. Syst. Mag.

[15] Dormido S (2004). Control Learning: Present and Future. IFAC Annual Rev. Control.

[16] Sánchez J, Dormido S, Esquembre F (2005). The Learning of Control Concepts Using Interactive Tools. Comput. Appl. Eng. Educ.

[17] Esquembre F Easy Java Simulations.

[18] Esquembre F (2004). Easy Java Simulations: A Software Tool to Create Scientific Simulations in Java. Comp. Phys. Comm.

[19] Christian W (2007). Open Source Physics: A User’s Guide with Examples.

[20] Åström KJ, Häagglund T (2005). Advanced PID Control.

[21] Ogata K (2006). Modern Control Engineering.

[22] Dorf R, Bishop R (2004). Modern Control Systems.

